# Under the same roof: co-location of practitioners within primary care is associated with specialized chronic care management

**DOI:** 10.1186/1471-2296-15-149

**Published:** 2014-09-02

**Authors:** Juliet Rumball-Smith, Walter P Wodchis, Anna Koné, Tim Kenealy, Jan Barnsley, Toni Ashton

**Affiliations:** Institute of Health Policy, Management and Evaluation, University of Toronto, 155 College St, Suite 425, Toronto, ON M5T 3M6 Canada; Institute for Clinical Evaluative Sciences, Toronto, ON Canada; Department of General Practice and Primary Health Care, School of Population Health, University of Auckland, Auckland, New Zealand; Health Systems, School of Population Health, University of Auckland, Auckland, New Zealand

**Keywords:** Canada, New Zealand, Chronic disease, Patient care team, Primary care, Co-location

## Abstract

**Background:**

International and national bodies promote interdisciplinary care in the management of people with chronic conditions. We examine one facilitative factor in this team-based approach - the co-location of non-physician disciplines within the primary care practice.

**Methods:**

We used survey data from 330 General Practices in Ontario, Canada and New Zealand, as a part of a multinational study using The Quality and Costs of Primary Care in Europe (QUALICOPC) surveys. Logistic and linear multivariable regression models were employed to examine the association between the number of disciplines working within the practice, and the capacity of the practice to offer specialized and preventive care for patients with chronic conditions.

**Results:**

We found that as the number of non-physicians increased, so did the availability of special sessions/clinics for patients with diabetes (odds ratio 1.43, 1.25–1.65), hypertension (1.20, 1.03–1.39), and the elderly (1.22, 1.05–1.42). Co-location was also associated with the provision of disease management programs for chronic obstructive pulmonary disease, diabetes, and asthma; the equipment available in the centre; and the extent of nursing services.

**Conclusions:**

The care of people with chronic disease is the ‘challenge of the century’. Co-location of practitioners may improve access to services and equipment that aid chronic disease management.

**Electronic supplementary material:**

The online version of this article (doi:10.1186/1471-2296-15-149) contains supplementary material, which is available to authorized users.

## Background

Chronic diseases are the leading cause of death worldwide, and their burden is predicted to increase [1]. Governments of low- and high-income nations alike are focused on how best to provide systematic and comprehensive care for patients with chronic conditions, within their primary healthcare systems [1]. Canada and New Zealand (NZ) offer an interesting study. NZ provides a partially tax-funded primary healthcare system, wherein Primary Health Organisations (PHOs) are responsible for the delivery of primary care and preventive services for a defined population. The PHOs receive capitation-based funding from central government according to the size, demographics, and health needs of the enrollees. PHOs in turn contract with a network of general practitioners (GPs) and other service providers, with most patients contributing co-payments for GP consultations [2]. Canada’s territories and provinces also provide primary care through private providers. However, services are largely paid for by government on a fee-for-service basis, and public funding ensures essentially free access at the point of care. Each province in Canada operates a distinct health system. Ontario is the largest province with primary care provided by a number of different organizational primary care models, including enhanced fee-for-service and capitation-based payment practices [2].

Both countries are similarly affected by the growing burden of chronic disease. More than 60% of NZ adults have been diagnosed with a long-term condition [3], and 70% of Canadians aged 45 years or over have 2 or more chronic diseases [4, 5]. As expected, the impact on the primary care system is substantial –80% of adult visits to GPs in Canada are due to chronic condition management [6]. However, delivering specialized and systematic chronic disease care requires time and skills not necessarily within the capacity of the GP: Ostbye et al. estimated physicians required more than 3 hours per day to practice evidence-based care for each well-controlled chronic condition [7].

Patient care teams may be an efficient means of providing systematic, safe and best practice care for complex patients [8], and are widely encouraged by researchers [8, 9] and international bodies [10, 11]. Their promotion reflects both the published evidence for their effectiveness, as well as their place in theoretical models of chronic care [12, 13]. While introducing other providers may risk care fragmentation, proponents state that interdisciplinary care is patient-centered and more comprehensive [9, 14, 15], improves concordance with clinical guidelines [16], and has positive effects on both job satisfaction and skill development for practitioners, and patient satisfaction and self-care skills [17]. Systematic reviews and meta-analyses have shown improved clinical outcomes in team-based primary care for people with chronic conditions such as diabetes [18], depression [19], and hypertension [20].

The health policies of Canada and NZ both emphasize the role of interdisciplinary care in the management of chronic conditions [21, 22]. NZ’s Primary Health Care Strategy states, “*No single practitioner or type of practitioner can meet people’s needs completely. A range of practitioners with the skills to communicate and collaborate in the patient’s interest are needed*” [22]; Canadian policy directed providers to ensure access to multidisciplinary teams for at least 50% of the population by 2011 [23]. Despite these declarations, in a survey undertaken in 2006, these countries rated poorly in the use of patient care teams - out of seven Commonwealth countries, Canadian GPs reported the lowest rate of routine use of multidisciplinary teams in the management of patients with multiple chronic conditions (25%). The proportion of NZ respondents was higher at 57%, but still low compared to family physicians from other nations [24].

Although ‘interdisciplinary’ and ‘multidisciplinary’ are often used interchangeably, the terms are distinct. Whereas ‘multidisciplinary’ refers to the simple involvement of several types of professions in patient care, ‘interdisciplinary’ care requires a shared collaborative approach, “*mutually respectful engagement between health professionals in planning and implementing care together*” [25]. For this reason (and others [26]), research in this area is problematic, as a successful interdisciplinary team has both structural characteristics (such as the number of practitioners, their roles and locations) and interpersonal/process features, which may be difficult to assess. Accordingly, in this study we have focused on a discrete measurable construct– the co-location of multiple disciplines within the primary care practice.

Shared premises are thought to be a critical enabling factor for effective interdisciplinary care, to “*enhance information transaction, facilitate communication, and increase personal familiarity*” (p143 [27]). Co-location also reflects the growing interest in redesigning traditional primary care into ‘patient-centred medical homes’, such that there is one point of access to an array of services and professionals [14]. We hypothesized that primary care practices with co-located non-physician members may offer broader services and specialized care for patients with chronic conditions: such as more equipment, dedicated programs, and specific clinics tailored to their conditions. We used data from Quality and Costs of Primary Care in Europe (QUALICOPC) surveys in NZ and Ontario to explore the co-location of multiple disciplines in primary care centres, and the association of this factor with the provision of specialized care for people with chronic conditions.

## Methods

The QUALICOPC suite of four questionnaires was developed by the Netherlands Institute of Health Services Research following literature review, expert opinion and piloting. The suite was employed in 34 countries as part of a multi-national study on primary care performance [28]; researchers in each country made minor changes to the questionnaires to reflect local terminology and practices. We used items from the Practice and Physicians Experiences questionnaires, which explore access to services, medical record systems, funding arrangements, and interaction with specialists (among other areas). These surveys were completed by one GP in each practice.

### Sample

We aimed to achieve 220 participants from both NZ and Ontario, in accordance with the protocol of the broader QUALICOPC project [28]. Networks from the Ontario College of Family Physicians and the Centre for Effective Practice were used to contact potential participants in Ontario; those who expressed a willingness to participate were sent survey packs (n = 229) from which we received 184 completed questionnaires. A different approach was taken for the NZ sample: practices listed on registers held by the Royal New Zealand College of General Practitioners and the University of Auckland, plus additional practices identified from telephone books, were sent survey packs (n = 1373). From this mail-out and subsequent follow-up mail-outs, we received 168 responses, giving a combined total sample of 352 practices. Ontario physicians received a CAN$200 incentive to participate, while NZ physicians were entered into a draw to win one of five iPads. We obtained ethical approval for this study from the Health Sciences Research Ethics Board (University of Toronto, Ontario) and the University of Auckland Human Participants Ethics Committee (NZ).

### Measures

Our exposure variable was the co-location of non-physician disciplines, created from the response to a single survey item: “Which of the following disciplines are working in your practice/centre?” with 12 options: receptionist/medical secretary; practice nurse; community/home care nurse; psychiatric nurse; nurse practitioner; assistant for laboratory work; manager of the centre or practice (not a physician); midwife; physiotherapist; dentist; pharmacist and social worker. The NZ survey offered two additional options (psychologist and community health worker) but as these were not included in the Ontario questionnaire, responses to these options were not included in our data. Each NZ participant selected ‘yes’ if any of these disciplines were present; in Ontario the respondents indicated negatively if the discipline was not present and provided the number of full-time equivalent staff if the discipline was present. Giving each affirmative (any positive FTE count for Ontario) response a score of 1, we created a summative ordinal variable to represent the total number of disciplines in each practice (the exposure variable), providing a theoretical response range of 0–12.

Other independent variables included the proportion of the roster estimated by the respondent to be elderly, and the proportion estimated to be socially disadvantaged. These variables used the items “To what extent do you think your practice population compares to the average national level with respect to those over 70 years?” (in the former case), or “…with respect to those who are socially disadvantaged?” These questions had four possible responses: 1 = below average, 2 = average, 3 = above average, and 4 = don’t know. We also included a term to represent the number of enrolled patients or ‘roster size’. The observed data suggested that a small number of GPs misinterpreted this question, providing the number of patients for the entire practice instead of those for which they were personally responsible for, an error found in both Ontario and NZ responses. We employed country-specific tertiles to minimize this bias (other categorizations and definitions were also explored, with no substantive differences to the effect estimates of the principal findings). Finally, analyses of the total sample included a country variable.

We explored four sets of outcome variables that we hypothesized would be impacted by the presence of multiple co-located disciplines:

1. *Disease management programs*. The survey asked four questions with the stem “In the past 12 months, have you been involved in a disease management program…” “…for patients with chronic heart failure (CHF); asthma; chronic obstructive pulmonary disease (COPD); diabetes”. These types of program may increase the capacity of patients to self-manage their condition, facilitate monitoring of clinical state, and provide systematic coordination of complex cares. These four 0/1 dichotomous variables were investigated as individual outcomes.
2. *Special sessions*. Participants were asked “In the past 12 months, have you offered special sessions or clinics for: diabetic patients; hypertensive patients; the elderly” with a yes/no response format for the three options. These types of clinics/sessions allow patients regular access to care–before the time of a health crisis–as well as facilitate the monitoring of medication, biochemical markers and health status. They also enable specialist input within a primary care setting. These items were explored as dichotomous outcomes.
3. *Extent of nurse service provision*. This variable was created from responses to four items with the same stem: “Does your practice nurse or assistant independently provide… health promotion; routine checks of chronically ill patients; minor procedures (e.g. wound treatment); immunization”. Participants could indicate yes or no (1/0). This variable was investigated as a dichotomized variable, comparing ‘high-level’ (practices responding affirmatively for all 4 options) with the remainder (those indicating provision in 3 or less of the options). We hypothesized that increased nurse involvement is a critical component of accessible and specialized care for patients with complex and chronic conditions.
4. *Equipment in practice*. Participants were asked to indicate whether a specified piece of equipment (30 options e.g. ophthalmoscope–see Additional file 1 for the full list of options) was used in the practice; the positive responses were summed to create a linear ‘equipment score’ variable. The extent of equipment available in a practice is assumed to reflect its intention and capacity to be able to respond clinically to a broad range of presentations; to allow early management of deteriorating patients and reduce unnecessary referrals.

### Statistical analysis

Unadjusted means/proportions were calculated to compare sample characteristics (roster size and demographics, number of co-located non-physician disciplines) in Ontario and NZ. For the multivariable analyses, we employed logistic regression models for all dichotomous outcome variables: special sessions for diabetic, hypertensive, elderly patients; disease management programs for people with CHF, asthma, COPD, or diabetes; and high-level nurse service providers. Linear regression models were used for the ordinal equipment score variable. Each model included the same set of covariates: roster size, estimated proportion of rostered population aged over 70 years, the proportion of roster perceived as socially disadvantaged, and country. We used a complete case analysis approach; once respondents with missing items or “don’t know” responses were excluded, the sample size for each set of analyses ranged from 294 to 325. All statistical analyses were carried out with the statistical software Stata, version 13.1.

## Results

Table 1 provides the descriptive statistics of the sample. Ontario GPs reported on average lower roster sizes than that of NZ. NZ practices estimated that they saw more elderly patients (‘above average’ proportion reported by 49% of NZ respondents compared to only 34% of Ontario participants), and were more likely to perceive their patients as being socially disadvantaged. The respondents in both countries reported on average around four non-physician disciplines working at their practice, the frequencies of individual disciplines are given in Additional file 2 (Table 2).

**Table 1:** **Characteristics of study sample by country**

	Ontario	New Zealand	Total
N	167	163	330
**Rostered population**			
Mean (SD)	1653 (1423.8)	2569 (3057.6)	2105 (2416.0)
Median (min-max)	1400 (300–15000)	1800 (250–25000)	1500 (250–25000)
**Proportion of roster estimated aged over 70 years, frequency (%)**			
Below average	22 (13.2)	37 (22.7)	59 (17.9)
Average	89 (53.3)	46 (28.2)	135 (40.9)
Above average	56 (33.5)	80 (49.1)	136 (41.2)
**Proportion of roster estimates socially disadvantaged, frequency (%)**			
Below average	49 (29.3)	67 (41.1)	116 (35.2)
Average	74 (44.3)	48 (29.5)	122 (37.0)
Above average	44 (26.4)	48 (29.5)	92 (27.9)
**Number of co-located non-physician disciplines**			
Mean (SD)	4.04 (1.98)	3.93 (1.63)	3.98 (1.81)
Median (min-max)	4 (1–9)	4 (1–8)	4 (1–9)

**Table 2:** **Distribution of outcomes by country**

Outcomes	Ontario	New Zealand	Total
	Practices offering service (%)		
**Disease management programs**	N = 164	N = 153	N = 317
Chronic heart failure	50 (30.5)	43 (28.1)	93 (29.3)
Asthma	51 (31.1)	52 (34.0)	103 (32.5)
Chronic obstructive pulmonary disease	65 (39.6)	57 (37.3)	122 (38.5)
Diabetes	111 (67.7)	102 (66.7)	213 (67.2)
**Special sessions/clinics**	N = 165	N = 160	N = 325
Diabetes	74 (44.9)	89 (55.6)	163 (50.2)
Hypertension	30 (18.2)	37 (23.1)	67 (20.6)
Elderly	31 (18.8)	39 (24.4)	70 (21.5)
	N = 137	N = 157	N = 294
**High-level of nurse service provision**	61 (44.5)	143 (91.1)	204 (69.4)
	**Mean, SD (median)**		
	N = 167	N = 163	N = 330
Extent of equipment in practice*	12, 3.1 (12)	17.5, 2.1 (18)	14.7, 3.8 (15)

*Derived from summed variables, 1 point given for each affirmative response, maximum possible =30.

The distribution of responses to the availability of disease management programs was similar in Ontario and NZ. Slightly more NZ practices offered special sessions or clinics for all three groups of patients (16% compared to 13% of Ontario practices); 55% of Ontario practices did not provide this service for any of the groups (compared to 43% in NZ). Most NZ practices indicated a high level of nurse service provision, 143 (91%) selected ‘yes’ to all four of these items, however only 45% of Ontario practices reported the same level of provision. The amount of equipment available also differed, on average Ontario practices had 12 of the 30 items while NZ practices had a mean equipment score of 17.5 (Table 3).

**Table 3:** **Effect estimates (odds ratios/beta and 95% confidence intervals) of the association between the count of co-located non-physician disciplines and outcomes reflecting specialized chronic care management**

Independent variables	Unadjusted	+Practice size and demographics	+ Country
	**Odds ratios (95% CI)**		
**Disease management programs**			N = 317
Chronic heart failure	1.09 (0.96–1.25)	1.08 (0.95–1.24)	1.08 (0.95–1.24)
Asthma	1.18 (1.03–1.34)	1.17 (1.03–1.34)	1.17 (1.03–1.34)
Chronic obstructive pulmonary disease	1.20 (1.06–1.36)	1.19 (1.05–1.36)	1.19 (1.05–1.36)
Diabetes	1.41 (1.21–1.63)	1.40 (1.20–1.63)	1.40 (1.20–1.63)
**Special sessions/clinics**			N = 325
Diabetes	1.41 (1.23–1.61)	1.42 (1.24–1.62)	1.43 (1.25–1.65)
Hypertension	1.16 (1.00–1.34)	1.19 (1.03–1.38)	1.20 (1.03–1.39)
Elderly	1.19 (1.03–1.37)	1.21 (1.05–1.40)	1.22 (1.05–1.42)
			N = 294
**High level of nurse provision**	1.19 (1.03–1.39)	1.19(1.03–1.39)	1.39 (1.16–1.66)
	**Beta (95% CI)**		N = 330
**Equipment score**	0.64 (0.43, 0.86)	0.65 (0.43, 0.87)	0.69 (0.55, 0.83)

The multivariable analyses revealed consistently positive associations between the number of co-located disciplines and the outcomes investigated. In analyses of the total sample, positive significant associations were observed for all of the disease management programs, with the exception of the CHF outcome, which did not reach statistical significance at the 95% confidence level. As the number of disciplines in the practice increased by one, the odds of participating in programs for diabetes increased by 40%, asthma by 17%, and COPD by 19%. In the analyses investigating the likelihood of special sessions for patients with diabetes, hypertension or the elderly, the odds ratio (OR) ranged from 1.20–1.43, all were statistically significant at the 95% confidence level. A positive association between the co-location of disciplines and ‘high-level’ nurse service provision (OR 1.39 95% CI 1.16–1.66), and the amount of equipment available at the practice (beta 0.69 95% CI 0.55, 0.83) was also found.

When the data were analyzed by country (data not shown, see Additional file 3), there were some differences in the effect size between the two jurisdictions. The largest of these related to the analysis of nurse service provision: the OR for the Ontario respondents was 1.33 (1.10–1.62) compared to 1.97 (1.12–1.46) in NZ. The country variable was a key covariate in several of the analyses (special sessions for patients with diabetes, equipment score, and extent of nursing services), as evidenced by a positive OR or beta, significant at the 95% confidence level. However, in all of the country-specific analyses, there were substantial overlaps in the 95% confidence levels surrounding the estimates, and it is difficult to draw conclusions when comparing the two jurisdictions.

## Discussion

In our sample of 330 General Practices from Ontario and NZ, we find positive associations between the number of co-located disciplines and the provision of special sessions for people with diabetes, people with hypertension, and for the elderly; participation in disease management programs for diabetes, COPD, and asthma; the amount of equipment used within the practice; and the extent of nurse service. These findings support the hypothesis that the co-location of multiple disciplines within a primary care practice is associated with their capacity to provide broad, specialized and preventive care for people with chronic disease.

This study has limitations, one of which is its low response rate. Participation was voluntary, and recruitment was not pursued once more than 220 eligible practices had indicated a willingness to participate (in accordance with the protocol of the wider multi-country study) [28], even though a number of practices did not return surveys that they had agreed to complete. This approach ensured adequate power for the analyses, but may have affected the generalizability of the estimates. There are insufficient supporting data to investigate the representativeness of the samples at this time, so we cannot estimate the impact of potential response bias. That said, there are few scenarios whereby the relationship between the number of co-located disciplines and the provision of specialized chronic care might differ according to a characteristic associated with participation. It is possible that non-respondent practices had (for example) higher numbers of co-located disciplines and fewer specialized clinics for patients with chronic conditions, or fewer co-located disciplines and more disease management programs. However, it is difficult to identify a practice characteristic that would be associated with both non-participation and either of these contradictory findings in sufficient strength to nullify the results observed. Additionally, the consistency of our findings with current theory around patient care teams and quality of care for patients with chronic conditions, and their congruence with the published literature supports their internal validity [9, 14, 15]. As such, these findings are informative and helpful in guiding future research in this area.

Other limitations of the study include the self-report nature of the data. However, given that the surveys consider current care arrangements only, substantial recall bias is unlikely. Second, as discussed, the exposure variable does not measure either the presence or success of interdisciplinary care. Nor can we conclude that patients with chronic conditions received benefit from the services/equipment offered by the practices, only that they had the capacity to provide this level of care. Third, there may be uncontrolled confounding by variables not included in the model. We explored the role of other possible confounders such as respondent age and sex, but found no evidence of any impact. However, it is possible that rurality may have a role–for example, rural practices may carry a wider range of equipment, reflecting their relative isolation from specialist and referral centres. That said, we found that the practice location (as defined by the QUALICOPC survey into urban, suburbs, small town, mixed urban-rural and rural) had little impact on the effect estimates for the associations of interest and was not a significant confounder. While there is a theoretical argument for including this variable in the analyses irrespective of its influence on the estimates, there may be differences in how the two jurisdictions interpret these labels of practice location, such that the QUALICOPC variable we have available may be a source of differential measurement error - on balance, a more parsimonious model was preferred. Organizational variables may also have an impact; there is evidence from Ontario that funding model is associated with the comprehensiveness of care [29]. While we do not have information about the PHO status of the NZ participants, it is also possible that being a member of a larger PHO with greater staff and financial resources may facilitate the capacity of the practice to provide more specialized services. Research involving these types of structural variables would help to explore how some practices were able to achieve their level of service provision within the current environment. Fourth, we excluded some participants because of missing or ‘don’t know’ data in key covariates and this may have affected the precision of our estimates.

We find that practices with a greater number of disciplines may offer more specialized and preventive care for people with chronic conditions. The implications for policymakers and funders seem clear, as currently GPs in these two jurisdictions are failing to consistently meet the needs of those with chronic conditions. A Commonwealth Fund survey of GPs in seven countries found that only 55% of the Canadian respondents indicated they were ‘well-prepared’ to provide optimal care for patients with multiple chronic conditions, the lowest percentage of all countries [24]. In NZ around 80% of all preventable deaths are due to suboptimal care of those with chronic disease [30]. Additionally, geographical access is a barrier to high quality chronic care in parts of NZ and Ontario [31, 32]. While it is possible that co-location may lend itself to a smaller number of larger practices (limiting geographical accessibility), the economies of co-location may also allow communities to sustain a broader range of practitioners than if they were operating independently. If implemented carefully, shared premises may be a strategy to improve care for these dispersed or isolated populations [33].

Organizational and funding constraints suggest the housing of multiple disciplines is beyond the capacity of many primary care practices. Multidisciplinary care in Ontario is highly dependent on the organizational model; while Family Health Teams and Community Health Centres prioritize service delivery by patient care teams, these types of practice are in the minority. In Canada nearly 50% of GPs operate from fee-for-service models, an approach that may disincentivize the delegation of tasks to non-physicians. The NZ National Health Committee also suggested funding was an administrative barrier to collaborative health care, in particular for those with multiple conditions, which may not individually satisfy criteria for targeted funding [30]. Additionally, the traditional culture of medical care delivery may be a factor in both countries. For example, despite substantial investment in education programs to improve teamwork in healthcare (in 2003, CAN$85 million was dedicated to interdisciplinary education in Canada), many Canadian GPs (including prominent professional organizations) resist increasing the responsibility held by non-physicians [34, 35].

This study shows a positive association between the co-location of non-physicians at primary care practices, and their capacity to offer enhanced services for patients with chronic conditions and a broader range of equipment. Research into interdisciplinary care is difficult–studies must consider how best to assess the presence of true ‘interdisciplinary team care’ [26], and select outcome variables carefully, as excellent primary care may both increase and decrease ‘adverse’ outcomes (such as acute hospital readmission [36]). That said, the optimal composition and organization of patient care teams is not known and research is required to identify the critical components. While some of the practitioners in this study reported their practice housed 9 distinct disciplines, it is possible that smaller teams can achieve more of the benefits of broader interdisciplinary care. There is evidence for the positive impact of nurse practitioners in chronic care, for example as part of supportive self-management programs [37]. NZ advisory statements echo this, concluding that practice nurses “*lead the delivery of proactive care for people with long term conditions*”(p22/23 [38]). Given their small numbers in NZ, and a relative decline in the per capita ratio of nurse practitioners in Canada, research into nursing roles is particularly pressing [39]. Community pharmacists may also be key contributors, [40, 41], as may community health workers, social workers or pension/benefit liaison officers. A desire for co-located social and health services was a dominant theme of feedback from NZ people with chronic conditions [30]; this approach has been trialed in primary care in other countries such as Sweden and England [42].

## Conclusion

The co-location of multiple disciplines may be a means to facilitate the delivery of specialized and preventive care services for people with chronic conditions. We suggest policy-makers and health care providers review how funding and organizational arrangements may enable this primary care structure. Research into the optimal size and composition of patient care teams would help to identify the critical roles for successful interdisciplinary care.

## Electronic supplementary material

Additional file 1: **Options able to be selected in response to the item “Indicate the equipment used in your practice by yourself or your staff (mark all that apply)”.** (DOCX 15 KB)

Additional file 2: **Frequency of co-located disciplines in Ontario and New Zealand samples.** (DOCX 15 KB)

Additional file 3: **Effect estimates (odds ratios/beta and 95% confidence intervals) of the association between the count of co-located non-physician disciplines and outcomes reflecting specialized chronic care management, according to country.** (DOCX 15 KB)

## Abbreviations

NZ: New Zealand
PHO: Primary Health Organisation
CHF: Chronic Heart Failure
CI: Confidence Interval
COPD: Chronic Obstructive Pulmonary Disease
GP: General Practitioner
OR: Odds Ratio
QUALICOPC: Quality and Costs of Primary Care in Europe.
