# Supplementary material for: Under the same roof: co-location of practitioners within primary care is associated with specialized chronic care management
Source: BMC Fam Pract. 2014 Sep 2;15:149. doi: 10.1186/1471-2296-15-149 (PMC4171578; doi:10.1186/1471-2296-15-149)
Supplement: Supplementary file 1 — Additional file 1: Options able to be selected in response to the item “Indicate the equipment used in your practice by yourself or your staff (mark all that apply)”. (DOCX 15 KB) [file 12875_2014_1124_MOESM1_ESM.docx]

**Additional file 1**

Options able to be selected in response to the item “Indicate the equipment used in your practice by yourself or your staff (mark all that apply)”.

| Hemoglobinometer |
| --- |
| Any blood glucose test set |
| Any cholesterol meter |
| Blood cell counter |
| Ophthalmoscope |
| Proctoscope |
| Otoscope |
| Gastroscope |
| Sigmoidoscope |
| X-ray |
| Ultrasound for abdomen/fetus |
| Microscope |
| Audiometer |
| Bicycle ergometer |
| Eye tonometer |
| Peak flow/PEF meter |
| Spirometer |
| Electrocardiograph |
| Blood pressure meter |
| Infusion set |
| Doctor’s bag for emergencies and home visits |
| Urine catheter |
| Coagulometer (Point of care INR) |
| Set for minor surgery |
| Suture set |
| Defibrillator |
| Disposable syringes |
| Disposable gloves |
| Refrigerator for medicines |
| Resuscitation equipment |
