# Supplementary material for: Under the same roof: co-location of practitioners within primary care is associated with specialized chronic care management
Source: BMC Fam Pract. 2014 Sep 2;15:149. doi: 10.1186/1471-2296-15-149 (PMC4171578; doi:10.1186/1471-2296-15-149)
Supplement: Supplementary file 2 — Additional file 2: Frequency of co-located disciplines in Ontario and New Zealand samples. (DOCX 15 KB) [file 12875_2014_1124_MOESM2_ESM.docx]

**Additional file 2**

Frequency of co-located disciplines in Ontario and New Zealand samples.

| **Co-located disciplines** | **Ontario**  **(n=167)** | **New Zealand**  **(n=163)** |
| --- | --- | --- |
| **N (%)** |  |  |
| Receptionist/medical secretary | 166 (99.4) | 163 (100) |
| Practice nurse | 122 (73.1) | 160 (98.2) |
| Community/home care nurse | 13 (7.8) | 17 (10.4) |
| Psychiatric nurse | 3 (1.8) | 15 (9.2) |
| Nurse practitioner | 63 (37.7) | 24 (14.7) |
| Assistant for laboratory work | 40 (24.0) | 20 (12.3) |
| Manager of the centre or practice (not a physician) | 110 (65.9) | 120 (73.6) |
| Midwife | 1 (0.6) | 19 (11.7) |
| Physiotherapist | 20 (11.4) | 42 (25.8) |
| Dentist | 14 (8.4) | 12 (7.4) |
| Pharmacist | 63 (37.7) | 38 (23.3) |
| Social worker | 61 (36.5) | 10 (6.1) |
