# Supplementary material for: Under the same roof: co-location of practitioners within primary care is associated with specialized chronic care management
Source: BMC Fam Pract. 2014 Sep 2;15:149. doi: 10.1186/1471-2296-15-149 (PMC4171578; doi:10.1186/1471-2296-15-149)
Supplement: Supplementary file 3 — Additional file 3: Effect estimates (odds ratios/beta and 95% confidence intervals) of the association between the count of co-located non-physician disciplines and outcomes reflecting specialized chronic care management, according to country. (DOCX 15 KB) [file 12875_2014_1124_MOESM3_ESM.docx]

**Additional file 3**

Effect estimates (odds ratios/beta and 95% confidence intervals) of the association between the count of co-located non-physician disciplines and outcomes reflecting specialized chronic care management, according to country.

| **Independent variables** | | **Ontario** | **New Zealand** |  |
| --- | --- | --- | --- | --- |
|  |  |  |  |  |
|  | | **Odds ratios (95% CI)** | | |
| **Disease management programs** | | N=164 | N=153 | |
| Chronic heart failure | | 1.07 (0.90 – 1.27) | 1.08 (0.87 – 1.35) | |
| Asthma | | 1.18 (0.99 – 1.40) | 1.18 (0.95 – 1.46) | |
| Chronic obstructive pulmonary disease | | 1.20 (1.02 – 1.42) | 1.16 (0.94 – 1.43) | |
| Diabetes | | 1.46 (1.20 – 1.78) | 1.34 (1.05 – 1.71) | |
| **Special sessions/clinics** | | N=165 | N=160 | |
| Diabetes | | 1.46 (1.22 – 1.74) | 1.44 (1.14 – 1.80) | |
| Hypertension | | 1.41 (1.13 – 1.76) | 1.00 (0.79 – 1.27) | |
| Elderly | | 1.18 (0.96 – 1.45) | 1.26 (1.01 – 1.58) | |
|  | | N=137 | N=157 | |
| **High level of nurse provision** | | 1.33 (1.10 – 1.62) | 1.97 (1.12 – 1.46) | |
|  | | **Beta (95% CI)** |  | |
| **Equipment score** | N=167 | | N=163 | |
|  | 0.70 (0.49, 0.92) | | 0.68 (0.51, 0.85) | |

Model includes terms for roster size, the proportion of patients estimated to be aged over 70 years, and the proportion of patients estimated to be socially disadvantaged.
